# Nociception level index variations in ICU: curarized vs non-curarized patients — a pilot study

**DOI:** 10.1186/s44158-024-00193-z

**Published:** 2024-08-20

**Authors:** Emilio Bonvecchio, Davide Vailati, Federica Della Mura, Giovanni Marino

**Affiliations:** ICU and Anaesthesia Department, Melegnano Hospital-ASST Melegnano and Martesana, Vizzolo Predabissi, Milan, Italy

**Keywords:** Nociception, Pain monitoring, ICU pain, Goal-directed pain treatment

## Abstract

**Purpose:**

Pain is a major physiological stressor that can worsen critical medical conditions in many ways. Currently, there is no reliable monitoring tool which is available for pain monitoring in the deeply sedated ± curarized critically ill patients. This study aims to assess the effectiveness of the multiparameter nociception index (NOL®) in the critical care setting. We compared NOL with traditionally used neurovegetative signs and examined its correlation with sedation depth measured by bispectral index (BIS®) electroencephalographic (EEG) monitoring.

**Methods:**

This retrospective monocentric cohort study was conducted in a general intensive care unit, including patients who required moderate-to-deep levels of sedation with or without continuous neuromuscular blockade. The performance of NOL was evaluated both in the entire studied population, as well as in two subgroups: curarized and non-curarized patients.

**Results:**

NOL demonstrated greater accuracy than all other indicators in pain detection in the overall population. In the non-curare subgroup, all indices correctly recognized painful stimulation, while in the patients subjected to neuromuscular blocking agent’s infusion, only NOL properly identified nociception. In the former group, EEG’s relation to nociception was on the border of statistical significance, whereas in the latter BIS showed no correlation with NOL.

**Conclusion:**

NOL emerges as a promising device for pain assessment in the critical care setting and exhibits its best performance precisely in the clinical context where reliable pain assessment methods are most lacking. Furthermore, our research confirms the distinction between sedation and analgesia, highlighting the necessity for distinct monitoring instruments to accurately assess them.

## Introduction and objectives

Pain assessment in critically ill patients remains a significant clinical challenge [1–4]. Periprocedural pain is frequently reported, with a variable proportion ranging from 30 to 80% in this population, particularly during procedures such as arterial line insertion, chest tube and drain removal, which are considered the most painful for intensive care unit (ICU) survivors [5, 6]. Other procedures, including mechanical ventilation, tracheal suctioning, physiotherapy, nursing (i.e. changes in patient’s position, mobilization, hygiene and wound or burn dressing changes), have also been identified as sources of discomfort and pain [5].

The scope of the problem is substantial with more than half of ICU patients experiencing moderate-to-severe pain at rest and 80% reporting pain during procedures [7–10].

Of even greater concern is the fact that the incidence and prevalence of pain in the critical setting have shown minimal variation over the past decades, despite advancements in therapeutic options. More than 15 years ago, the DOLOREA study revealed that over 30% of mechanically ventilated patients reported a painful experience [9]; recent studies like the cross-sectional, multicentre, multinational Europain® study [5] or systematic reviews like the one by Nordness et al. [1] have confirmed these discouraging data, showing that pain epidemiology in the critical care setting has been basically unchanged since.

Untreated pain amplifies the stress response and causes increased myocardial oxygen consumption, rise in catecholamines levels, systemic release of inflammatory mediators, increased energetic metabolism, hyperglycaemia, immunosuppression, impaired wound healing and hypercoagulability [11]. Poorly managed pain from abdominal incisions reduces diaphragmatic function, causing hyperventilation and atelectasis. Severe pain disrupts the coupling of brain and ventilation metabolic curves, with impact on respiratory drive [12]. Inadequate pain relief in sedated patients may result in agitation, delirium with psychological sequelae like post-traumatic stress disorder, depression and anxiety or progression to chronic pain [11, 13]. The presence of on-going and persistent pain has been proven to cause increased length of ICU stay and prolonged mechanical ventilation [9, 13].

Pain assessment in ICU patients usually cannot rely on self-report nor, frequently, on behavioural scales, so that the evaluation of pain is left to monitoring of neurovegetative signs variation and clinical judgement, both of which have proved to be untrustworthy [14]. Pain evaluation notably appears as a blind spot in the sickest cluster of patients, namely deeply sedated patients needing continuous neuromuscular blockade [15, 16]. The failure of pain monitoring in this population creates a paradox: pain cannot be properly assessed in the subgroup of patients who are likely prone to experiencing the most significant pain and painful manoeuvres, given the utmost severity of their clinical conditions.

On the other hand, deeply sedated patients do not experience pain in its entirety: hypnotic drugs generate a pharmacologically induced state of unconsciousness, so the transduction and elaboration of pain by cortical superior centres are absent.

Nonetheless, the response to tissue injury translates into a series of autonomic and biohumoral reactions [17]. Nociception refers to the neural processes of encoding and processing noxious stimuli, while pain refers to the subjective experience of actual or impending harm [18].

Though nociceptive stimulation usually leads to pain, pharmacological and brain lesion research demonstrated that one can exist without the other [19]. Several human neuroimaging studies support this finding, showing that pain and noxious stimulation intensity are dissociable at the level of brain activity [20, 21].

The neural circuitry for nociception is mainly located in the spinal cord without going up to the brain, although in certain circumstances the brain activity can suppress or amplify nociception responses. Unlike nociception, pain is a perception that requires functional brain activity. When the nociceptive signals are sent from the spinal cord neurons to the brain to produce unpleasant sensory and emotional experience, pain perception occurs. Although nociception usually produces pain perception, pain can occur without nociception, and nociception may not lead to pain. For example, when a patient under general anaesthesia responds to surgical stimulation as increased heart rate and blood pressure, it is a nociception, not pain. On the other hand, a patient with central neuropathic pain after stroke can experience pain without any nociception (https://pain.ucsf.edu/understanding-pain-pain-basics/nociception-versus-pain).

Pain is always a personal experience, influenced to varying degrees by biological, psychological and social factors. Therefore, it cannot be assessed in an objective manner, whereas nociception has theoretically the potential to be evaluated and quantified [22].

Given the challenges in assessing pain in critically ill patients and the strong demand for effective pain monitoring tools, there has been significant interest in new technologies developed for nociception monitoring in the ICU setting [19]. The ability to isolate the physical aspect of pain, separating it from the experiential nature, provides an opportunity to quantify pain and facilitate treatment through goal-directed protocols. Nociceptive monitoring tools are particularly useful for deeply sedated patients, as they allow for the evaluation of the pathophysiological response to pain and express it in semiquantitative terms.

Most of the newly developed electrophysiological devices for monitoring nociception and related pain in situations where self-reporting or behavioural pain tools cannot be utilized rely on measuring physiological markers associated with sympathetic-parasympathetic responses. These markers include pupillary dilation, heart rate variability HRV, and sudation [15].

One limitation of HRV-derived devices is their inability to account for the coupling between heart and respiration, which can be affected by factors such as cardiac arrythmia, pacemaker, bradypnea, medications and critical illness [15].

Among these various technologies available, the nociception level (NOL) index is a new multiparameter measure that has been developed based on the belief that combining multiple physiological parameters yields superior results compared to their individual use. The NOL index simultaneously integrates HRV, photoplethysmographic waveform amplitude, skin conductance and temperature. These parameters are then processed using a random forest algorithm to describe the level of nociceptive as an adimensional numerical value ranging from 0 to 100 [23].

NOL has already been extensively validated in the perioperative setting [24–31]. However, there is little guidance exists regarding the validity of NOL monitoring in the critically ill patients. Only two pilot studies investigated the use of NOL in the intensive care setting, but they were conducted on awake patients admitted to ICU after elective postcardiac surgery. These studies have shown that NOL can identify nociceptive stimuli and has been associated with Numerical Rating Scale (NRS) and Critical Care Pain Observation Tool (CPOT) scores [32, 33].

However, it should be noted that the postcardiac surgery intensive care is a specific and specialized context that may not be representative of the different realities within intensive care. Furthermore, both studies examined the performance of NOL in awake patients. The awake patient is not likely to be the optimal target of NOL monitoring, as by definition nociception refers to a purely organic process while the awake subject experiences the full range of emotions and sensations related to noxious stimulation. In the conscious patient, anxiety, fear and agitation can distort neurovegetative signs response, so that NOL values may not be indicative of the actual nociceptive state. Previous pilot studies, not including heavily sedated patients, did not account for a large proportion of ICU patients, who prove to be the main recipients of instrumental nociception monitoring.

Therefore, our first objective was to investigate the ability of the nociception level index to quantify nociception in this group of patients, namely adult patients who are unable to self-report due to moderate to deep sedation, with or without continuous neuromuscular blockade and are admitted to a general medical and surgical intensive care.

Our secondary goal was to evaluate the level of agreement between changes in NOL and corresponding variations in neurovegetative parameters during painful stimulation. While neurovegetative signs have traditionally been used as indicators of pain, this assumption has been widely questioned in recent years.

Thirdly, we aimed to elucidate the relation between NOL and the depth of sedation measured using processed electroencephalography. Arousal is frequently misunderstood as nociceptive activation, or, even worse, a deep state of hypnosis is wrongly assumed to indicate the absence of pain. Pain and sedation are two interrelated yet distinct elements of critical care, necessitating distinct approaches for assessment and monitoring. We hypothesized that the combined use of NOL and BIS could provide valuable information regarding the pathophysiological distinction between the two areas of sedation and analgesia and confirm the need for differentiated tools for monitoring these two entities.

## Material and methods

This observational retrospective monocentric cohort study was conducted in the general intensive care of the Melegnano Hospital, Milan.

The Ethics Territorial Committee of Lombardy 1 approved this research project on 20 March 2024 (CET 126–2024). This study was conducted in accordance with the Declaration of Helsinki. All patients signed both specific procedural information and informed consent, before or after the implementation of the study protocol, based on their clinical conditions at the time of enrollment.

All adult patients (age ≥ 18 years) admitted to the ICU from January to October 2022, who required moderate to deep levels of sedation with or without continuous neuromuscular blockade, were included in the analysis.

The exclusion criteria were as follows: major arrythmia not controlled by therapy, substantial vasopressor support (defined as norepinephrine infusion ≥ 0.1 mcg/kg/min or equivalents) and neuromuscular disease.

We deliberately employed broad inclusion criteria in order to obtain a sample that is as representative as possible of the real population routinely admitted to ICUs.

Contrary to other studies, patients receiving chronic *β*-blockers therapy were not excluded from our research. This decision was based on the validation by Bergeron et al. of the NOL index’s performance in this subset of patients [34].

All patients were continuously monitored using electrocardiography, invasive arterial pressure, and peripheral oxygen saturation.

Depth of sedation was assessed by bispectral index (BIS) monitoring. BIS value is a dimensionless number that ranges from 0, indicative of complete brain inactivity (i.e. isoelectric EEG), to 100, indicative of an awake and alert patient. BIS monitoring value range represents a continuum that correlates to the clinical state and expected responses of the brain state to the administration of sedative hypnotic drugs. Awake, unsedated, healthy individuals typically have BIS values above 90. With progressive deepening of the drug-induced sedative effects, the BIS value declines. A BIS threshold of 60 has a high sensitivity for identifying drug-induced unconsciousness. BIS values between 40 and 60 represent the target range identified by the manufacturer as adequate for general anaesthesia or the maintenance of a deep sedation level in curarized patients to avoid awareness. BIS values less than 40 represent a deeper hypnotic state deemed potentially harmful because of its relation with neurocognitive disorders, in particular delirium [3, 35, 36].

The level of sedation for each patient was also evaluated using the *Richmond Agitation Sedation Scale* (RASS), as per the unit protocol.

Nociception monitoring was performed using the NOL index supported by PMD200 platform. The NOL index is a single dimensionless number from 0 to 100 obtained through a noninvasive finger probe. Values above 25 are believed to be indicative of nociception and related pain. NOL between 0 and 25 represents an appropriately suppressed physiological response to noxious stimuli and suggests adequate analgesia. NOL below 10 for more than 1 min, during a painful stimulation, may indicate excessive anti-nociception and reduction of analgesics may be considered. The manufacture’s reference intervals were used: values above 25 were considered indicative of nociceptive excitation [4, 15, 22].

Pain presence and magnitude were also assessed for each patient using the *behavioural pain scale* (BPS), according to the unit protocol. When the behavioural scale was not applicable due to curarization or a deep level of sedation, the BPS was assigned the minimum score (*BPS* = 3).

For each patient, at least one baseline measurement was reported, which was defined as the absence of nociceptive stimulation in addition to the eventual condition of pain at rest.

Data were collected by medical and nursing staff of the ICU. The attending nurse or physician recorded the data. The source of data was the electronic medical record in use in our department: as per unit protocol BIS values, vital signs and BPS scale are regularly registered there. NOL data were noted separately by the charge nurse or physician indifferently.

Three routine procedures commonly performed in the ICU were selected as potentially painful manoeuvres: pronosupination, endobronchial aspiration and nursing (nursing was considered as patient mobilization, routinely hygiene manoeuvres and wound dressing changes).

Neurovegetative signs, sedation level assessed by means of the bispectral index and nociception level measured using NOL and BPS were recorded during each painful stimulation event in every studied patient.

The values included in the analysis were the median values in a 30-s interval before and after the nociceptive event. This approach is consistent with the methodological approach previously adopted by Edry, Sessler et al. in their preliminary intraoperative validation of NOL [25]. Unlike the operating room, where noxious stimulation is punctual and confined to surgery, critical care patients experience a constant background of pain on which nociceptive excitation is superimposed. Furthermore, in some cases, the reaction to stimulation is maximal but of brief duration, while more moderate but sustained responses can be clinically more relevant.

The sedation level was normalized on BIS values in order to make the results as independent as possible from the specific hypnotic agent used.

The duration of observation for each patient was set at 72 h. This timeframe was selected based on two reasons. Firstly, it was deemed appropriate to capture an adequate number of invasive manoeuvres and procedures for each patient. Secondly, it was considered sufficient to create a representative sample of the real critically ill population. This duration exceeds the typical ICU stay for patients admitted solely for postoperative monitoring or optimization of ongoing therapies, where a rapid recovery is anticipated.

### Statistical analysis

Two dichotomous conditions were defined for analysis: baseline, which refers to a state of functional rest without external nociceptive agents, and stimulation, which corresponds to painful manoeuvres or procedures included in the study protocol.

A mixed model for repeated measures was employed to assess the variation from the baseline to stimulation states.

For each parameter (BIS, NOL, HR, systolic blood pressure), a separate model was created with the dependent variable being the parameter and the independent variable being the presence or absence of stimulation.

To examine the relationship between the NOL index and the other indicators studied, a multivariate linear model was used. In this model, NOL served as the dependent variable, and BIS or neurovegetative signs, along with the presence or absence of stimulation, were considered as independent variables.

The sample was then stratified based on the presence or absence of neuromuscular blockade, and the same test was then applied to the resulting subgroups. The calculated *p*-value corresponds to the null *T*-test hypothesis, where *β* (the parameter estimate) is null. This indicates the absence of an association between the dependent and independent variable, accounting for the influence of other variables in the model.

Pearson’s correlation coefficient (r) was computed to evaluate the association between parameters under different painful stimuli.

## Results

A total of 22 patients were recruited. Among them, two were extubated before the 72-h observational time limit set by the study protocol. Two cases experienced issues with the photoplethysmographic signal quality of the NOL index, primarily due to detection problems with the finger probe in situations where there was reduced peripheral perfusion.

**Fig. 1:**
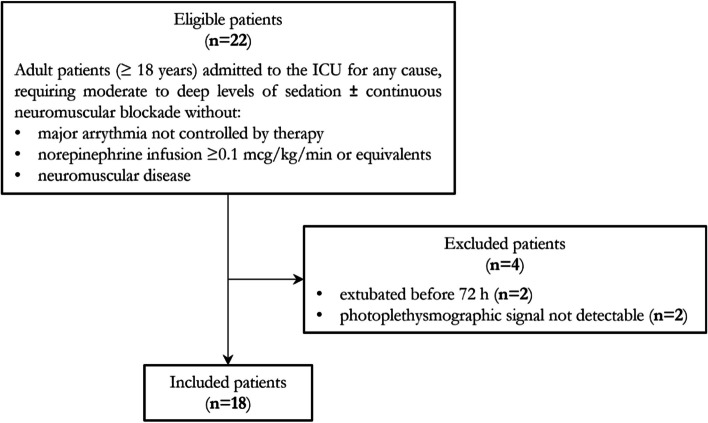
Flow chart for patient selection and inclusion and exclusion criteria for study

Therefore, a total of 18 patients were included in the final data analysis. Comprehensive data for all the include patients could be recorded, so there was no missing data flow chart (Fig. 1).

The demographic characteristics of the study participants are listed in Table 1.

**Table 1:** Demographic characteristics of the population studied

*General characteristics*	
N	18
Age	67.4 ± 9.68
Sex	M 12 (66.6%)
BMI	28.5 ± 4.8
***Admission cause***	
CARDS	7 (38.9%)
Poisoning	2 (11.1%)
Guillain-Barré syndrome	2 (11.1%)
Septic shock	7 (38.9%)
***Severity scores***	
SOFA at admission	8.5 (*IQR* 6)
SAPS II	56 (*IQR* 13)
***Hypnotic and analgesic drugs***	
Propofol	14 (77.8%)
Midazolam	7 (38.9%)
Dexmedetomidine	2 (11.1%)
Remifentanil	15 (83.3%)
Transdermic fentanyl	2 (11.1%)
***Other drugs***	
Neuromuscular blockade	6 (33.3%)
Vasopressor support (*norepinephrine*)	6 (33.3%)
***Pathological history***	
Arrythmias (*atrial fibrillation*)	4 (22.2%)
Pacemaker	1 (5.5%)
Ischaemic heart disease	3 (16.7%)
Arterial hypertension	9 (50%)
Peripheral vascular disease	4 (22.2%)
Diabetes type II	6 (33.3%)
Chronic lung disease	5 (27.8%)
Psychiatric disease	2 (11.1%)
Chronic pain therapy	0

*BMI* body mass index, *CARDS* COVID-19-associated ARDS, *SOFA* Sequential Organ Failure Assessment, *SAPS II* Simplified Acute Physiology Score, *IQR* interquartile Range

Data were collected at baseline and during nociceptive stimulation. The mean NOL index value at baseline was 10.92 ± 8.69. The mean NOL value during painful stimulation was 32.36 ± 18.07. The parameter distribution of the examined population is listed in Table 2.

**Table 2:** Parameters distribution of the studied population

		**BIS**	**NOL**	**BPS**	**HR (*bpm*)**	**SBP (*mmHg*)**
**Baseline**	N	64	64	64	64	64
	Mean value	50.00	10.92	3.33	80.11	123.52
	Standard deviation	13.96	8.69	0.67	18.94	22.28
**Stimulation**	N	97	97	97	97	97
	Mean value	60.14	32.36	3.70	84.55	133.06
	Standard deviation	18.72	18.07	1.07	19.77	24.93

*HR* heart rate, *bpm* beat per minute, *SBP* systolic blood pressure, *mmHg* millimetres of mercury

### Relationship between observed indicators and nociceptive stimulation

Statistically significant variations were found between baseline and stimulation for all the examined parameters using a mixed linear model for repeated measures (*p* < 0.05), as shown in Table 3. However, there were differences in the extent of deviation from baseline among the indicators.

**Table 3:** Mixed model for repeated measures

		**Beta**	**Standard error**	***p*-value**
BIS	Stimulation vs baseline	11.11	1.90	< 0.001
NOL	Stimulation vs baseline	21.60	2.24	< 0.001
BPS	Stimulation vs baseline	0.41	0.11	< 0.001
HR	Stimulation vs baseline	4.57	1.99	0.0214
SBP	Stimulation vs baseline	10.45	2.95	< 0.001

The effect of nociceptive stimulation is more accentuated for NOL (*β* = 21.6) and BIS (*β* = 11.11). Statistical significance was also more stringent for these measures (*p* < 0.0001). Neurovegetative signs, particularly heart rate, showed less variation compared to NOL and BIS, with *β* coefficients of 10.45 for SBP and 4.57 for HR. Interestingly, BPS exhibited the smallest changes, with a mean value at rest of 3.33 ± 0.67 and 3.7 ± 1.07 during painful stimulation (*β* 0.41).

### Analgesia-hypnosis correlation: analysis of the NOL-BIS relationship

**Fig. 2:**
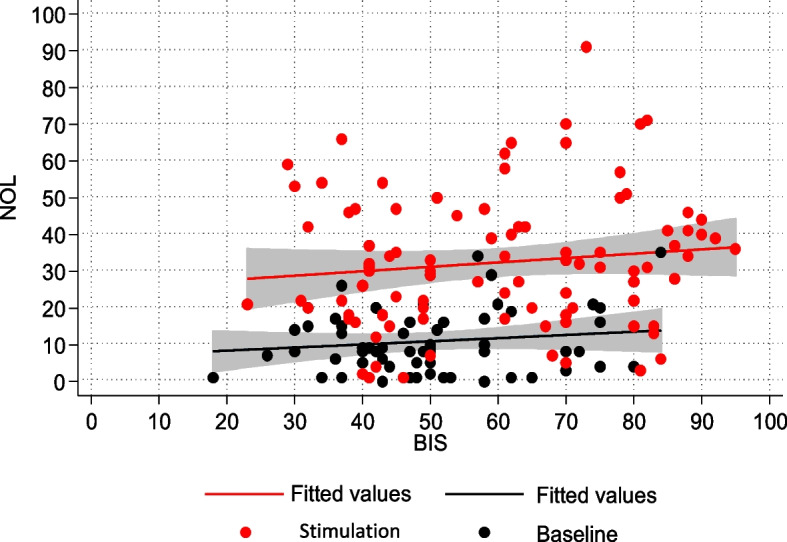
NOL-BIS relationship

The correlation between NOL and BIS was examined through a linear multivariate model to determine whether nociceptive stimulation led to changes in sedation depth. The regression model indicated no correlation between NOL and BIS (Fig. 2).

### Subgroup analysis: non-NMBA patients

To thoroughly investigate the relationship between NOL and the other indices, a further analysis was conducted by dividing the study population in two subgroups: patient under continuous infusion of neuromuscular blocking agents (NMBA) and patients who were not administered NMBA. These two populations represent two contrasting sedation settings: curarized patients underwent deep sedation regimen, while non-curarized patients were targeted for light-moderate sedation.

Table 4 displays the data distribution among the non-curarized patients. The mean value of BIS was 55.1 ± 13.95, indicating a moderate-light sedation level. RASS values hovered around − 2 to − 3, which is consistent with bispectral index recordings. BIS values increased to a mean value of 68.57 ± 17.05 during nociceptive stimulation.

**Table 4:** Parameters distribution in the non-NMBA subpopulation

		**BIS**	**NOL**	**BPS**	**HR (*bpm*)**	**SBP (*mmHg*)**
**Baseline**	N	41	41	41	41	41
	Mean value	55.10	11.12	3.51	74.15	123.00
	Standard deviation	13.95	9.69	0.78	15.81	23.13
**Stimulation**	N	61	61	61	61	61
	Mean value	68.57	31.52	4.11	80.80	133.28
	Standard deviation	17.05	17.76	1.17	18.74	26.03

*HR* heart rate, *bpm* beat per minute, *SBP* systolic blood pressure, *mmHg* millimetres of mercury

All the studied parameters exhibited a statistically significant variation from baseline during painful stimulation. However, it is important to note that the *β* coefficient related to NOL was superior to the *β* coefficient of all other examined indices.

**Fig. 3:**
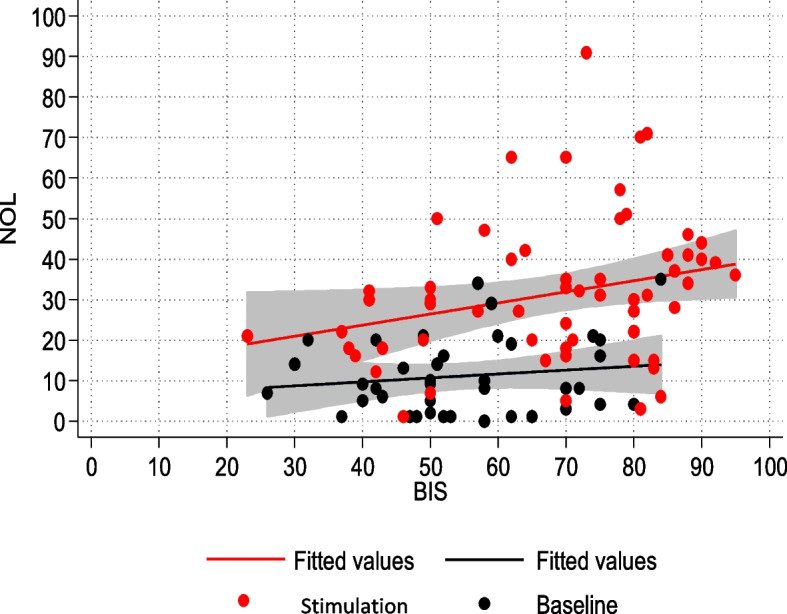
NOL-BIS relationship in the non-NMBA subpopulation

Next, the relationship between NOL-BIS and NOL-neurovegetative signs (HR, SBP) was investigated. The regression model demonstrated an association between NOL and BIS (Fig. 3). In the subpopulation of patients who were not subjected to NMBA infusion, nociceptive stimulation resulted in a statistically significant change in both NOL and BIS, although of different magnitudes.

**Fig. 4:**
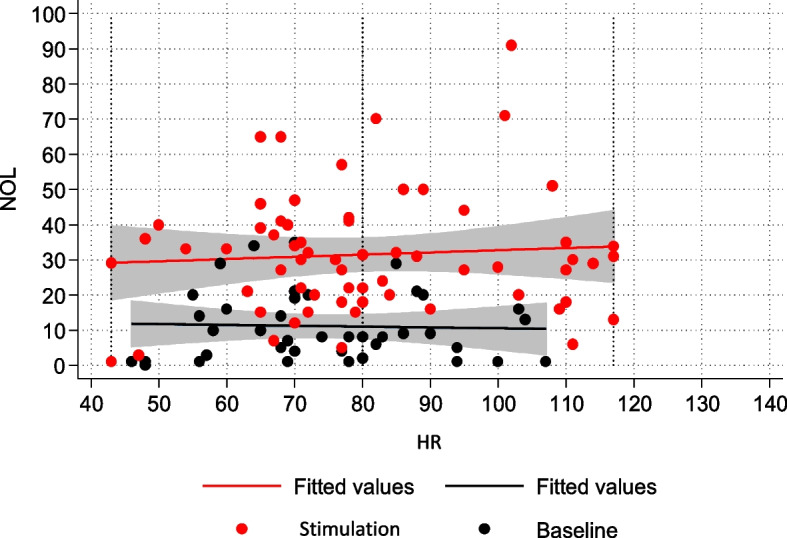
NOL-HR relationship in the non-NMBA subpopulation

**Fig. 5:**
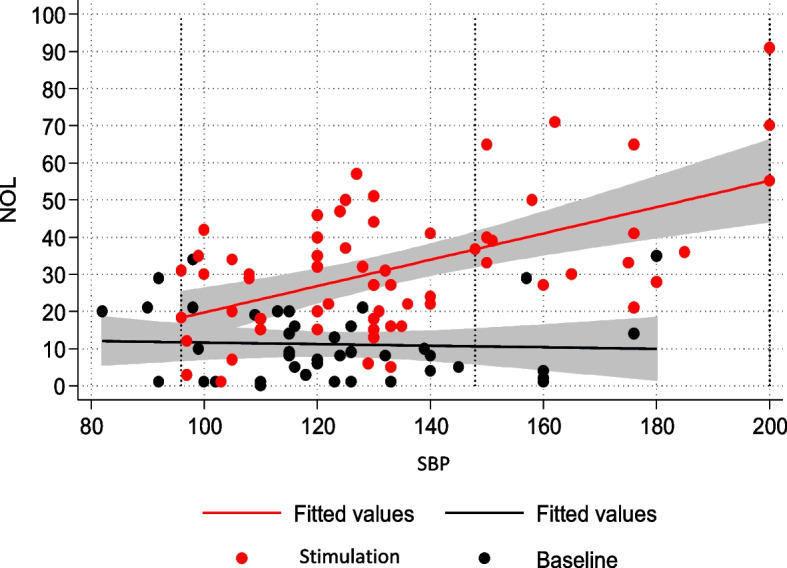
NOL-SBP relationship in the non-NMBA subpopulation

Likewise, a statistically significant correlation was observed between NOL variations and alterations in HR and SBP (Figs. 4 and 5).

### Subgroup analysis: NMBA patients

The first observation in the subpopulation receiving continuous NMBA infusion was that bispectral index values were lower compared to non-curarized patients, which is consistent with the requirement for deeper levels of sedation (Table 5). RASS value was − 5 for all patients in the NMBA subpopulation.

**Table 5:** Parameters distribution in the NMBA subpopulation

		**BIS**	**NOL**	**BPS**	**HR (*bpm*)**	**SBP (*mmHg*)**
**Baseline**	N	20	20	20	20	20
	Mean value	41.05	9.30	3.00	94.05	126.10
	Standard deviation	8.91	6.29	0.00	18.95	22.21
**Stimulation**	N	30	30	30	30	30
	Mean value	46.53	32.60	3.00	94.13	134.50
	Standard deviation	10.76	18.41	0.00	19.83	24.58

*HR* heart rate, *bpm* beat per minute, *SBP* systolic blood pressure, *mmHg* millimetres of mercury

The first notable difference in this data series is the lack of statistically significant changes in the neurovegetative parameters in response to painful stimulation. Therefore, neurovegetative signs did not appear to provide information about the nociceptive status of patients in this subpopulation. Data on the pain behavioural scale were not included since this scale is not applicable to pharmacologically paralyzed and deeply sedated patients.

Regarding variations in the bispectral index, a borderline statistical significance was observed (*P* = 0.048) (Table 6). It is possible that a larger sample size would have shown no association between BIS changes and painful excitation. The *β* coefficient relative to nociceptive BIS variation during painful stimulation is considerably lower than the NOL coefficient.

**Table 6:** Mixed model for repeated measures — NMBA patients

		**Beta**	**Standard error**	***p*-value**
BIS	Stimulation vs baseline	5.00	2.53	0.0480
NOL	Stimulation vs baseline	22.87	0.96	< 0.001
BPS	Stimulation vs baseline	N.a	N.a	N.a
HR	Stimulation vs baseline	0.60	3.95	0.8792
SBP	Stimulation vs baseline	8.35	4.67	0.0738

**Fig. 6:**
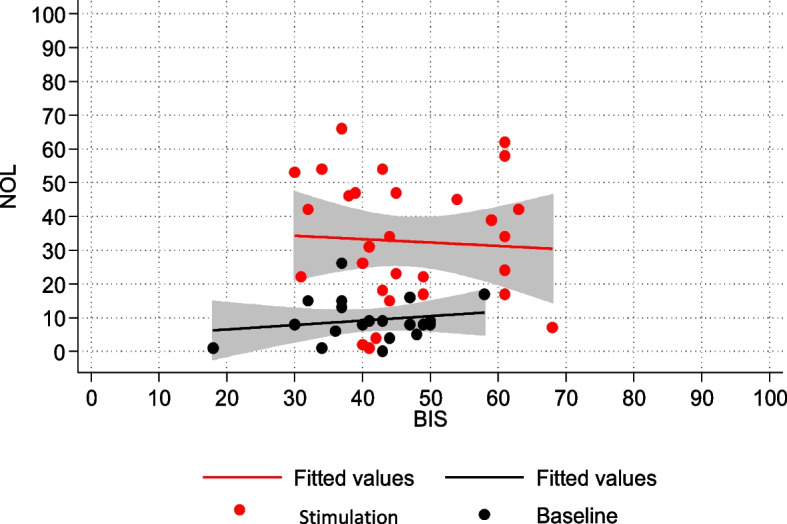
NOL-BIS relationship in the NMBA subpopulation

**Fig. 7:**
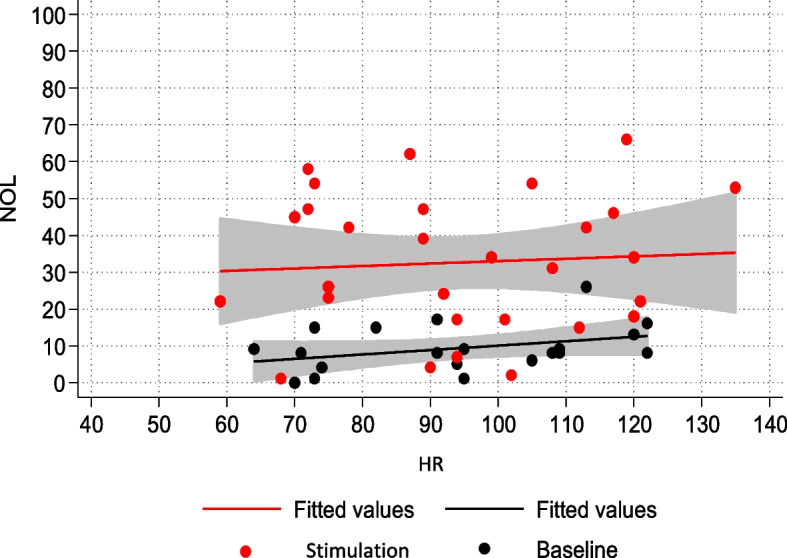
NOL-HR relationship in the NMBA subpopulation

**Fig. 8:**
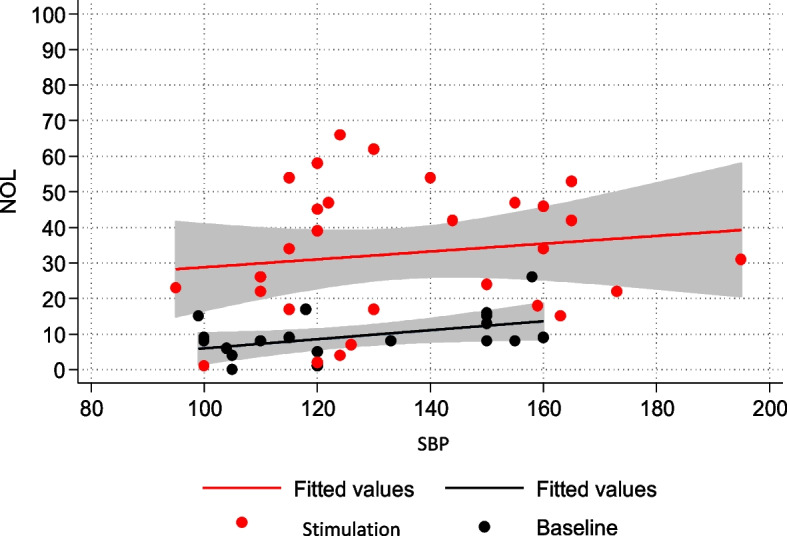
NOL-SBP relationship in the NMBA subpopulation

The relationship between NOL-BIS and NOL-neurovegetative signs was also examined in this subpopulation. However, no statistically significant association was found between NOL and bispectral index (Fig. 6), nor between NOL and neurovegetative signs (Figs. 7 and 8). In the subgroup of patients undergoing continuous neuromuscular blockade, neurovegetative indices do not exhibit any statically significant relationship with painful stimulation. This holds true for both their variation from baseline during nociceptive excitation and their correlation with NOL changes.

### Index variations according to different painful stimuli

To compare the response of the parameters to various nociceptive stimuli, a subanalysis was conducted considering three examined stimuli: endotracheal aspiration, nursing and pronosupination. Table 7 shows presents the distribution of parameters based on the different painful stimuli.

**Table 7:** Parameters distribution according to type of stimulation

		**BIS**	**NOL**	**BPS**	**HR (*bpm*)**	**SBP (*mmHg*)**
**Endotracheal aspiration**	N	62	62	62	62	62
	Mean value	60.97	29.31	3.71	85.24	134.34
	Standard deviation	20.15	16.87	1.09	20.80	23.28
**Nursing**	N	25	25	25	25	25
	Mean value	61.00	26.44	3.68	86.92	131.36
	Standard deviation	20.34	13.96	0.85	23.74	25.98
**Pronosupination**	N	7	7	7	7	7
	Mean value	44.43	40.86	3.00	86.43	127.71
	Standard deviation	9.76	21.06	0.00	20.29	11.51

*HR* heart rate, *bpm* beat per minute, *SBP* systolic blood pressure, *mmHg* millimetres of mercury

Pronosupination appeared to induce a significantly higher elevation in NOL compared to the other two stimuli, indicating a greater activation of the nociceptive pathway for this manoeuvre. The bispectral index for pronosupination was lower than that for endotracheal aspiration and nursing, likely due to the deeper level of sedation required for this manoeuvre.

**Fig. 9:**
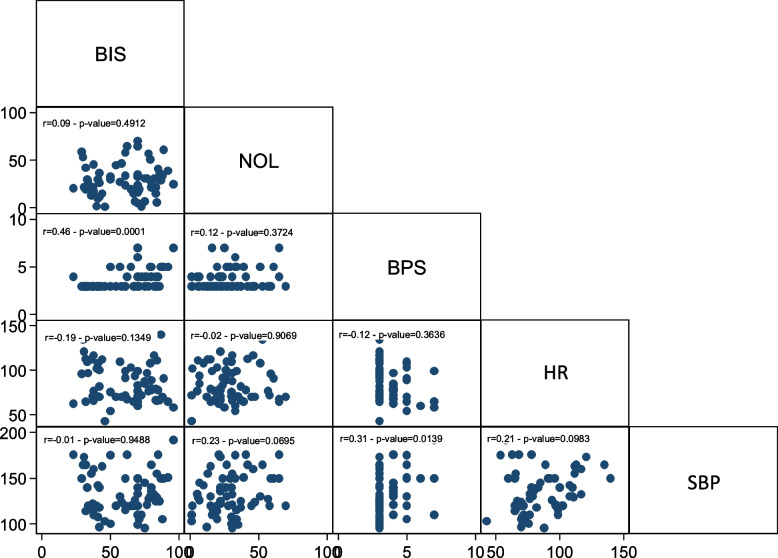
Endotracheal aspiration

**Fig. 10:**
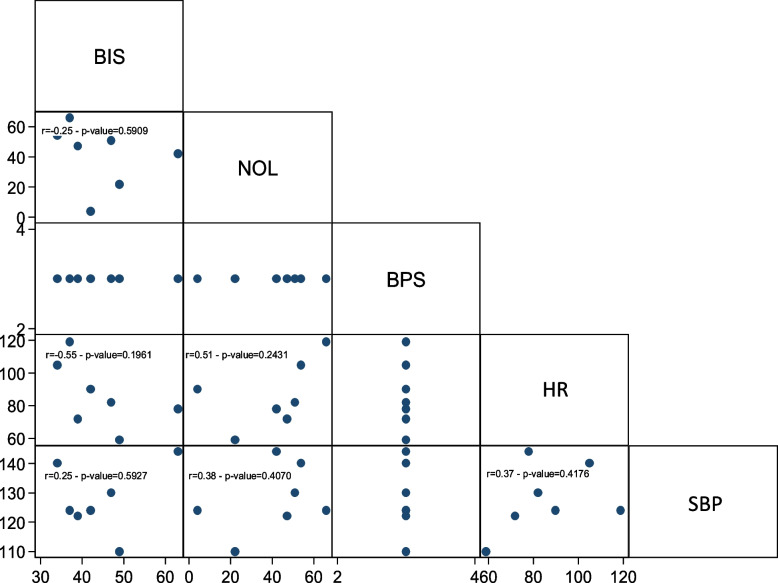
Pronosupination

**Fig. 11:**
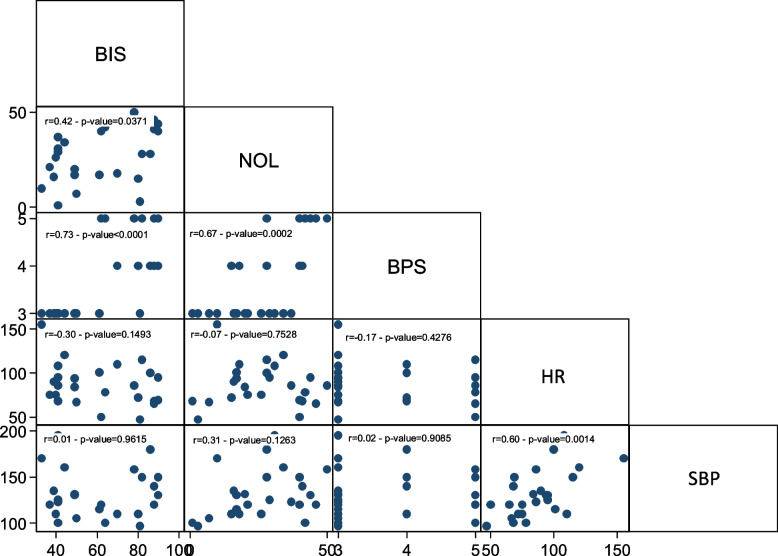
Nursing

None of the evaluated indices exhibited a statistically significant correlation with NOL during endotracheal aspiration. The same was true for pronosupination. Only nursing demonstrated an association between NOL and BPS, and BPS was also related to BIS during nursing (Figs. 9, 10 and 11).

## Discussion

To the best of our knowledge, this study, together with the ones conducted by Gélinas and Shahiri in the awake patients in the cardiac surgery ICU context, is one of the first to evaluate NOL in critically ill patients and the only one that considers its application in deeply sedated and continuously curarized patient in a general ICU setting.

In our studied population, we confirmed the ability of nociceptive stimuli to cause a significant and relevant increase in NOL. This finding is consistent with existing literature and expands the potential application of this device to general ICUs, where validation was previously lacking.

NOL, in fact, has already demonstrated to recognize and to quantify correctly the nociceptive response in various surgical settings during anaesthesia [24–31]. NOL was able to provide a scalar estimate of the degree of nociceptive response in relation to different opioid analgesic regimens [25, 26]. Nociception monitoring-guided anaesthesia was demonstrated to reduce postoperative pain scores in abdominal surgery during sevoflurane/fentanyl anaesthesia [24, 30]. NOL monitoring could also reduce opioid consumption, reducing well-known collateral effects associated with opioid overdosing during major abdominal surgery [29] and gynaecological laparoscopic surgery [31]. NOL monitoring also allowed better hemodynamic stability during major abdominal surgery [29] and gynaecological laparoscopic surgery [31].

NOL use in the ICU setting is still an area of research, as demonstrated by the two pilot studies by Gelinas and Shahiri [32, 33]. While taking into account the small population examined and their inherent limitations, these two studies offer an insight into the use of quantitative nociception monitoring in the intensive care setting. Both studies demonstrated that NOL can discriminate between nociceptive and non-nociceptive stimulations, and that NOL values are associated with measures of self-reported pain. As already outlined, the works of Gelinas and Shahiri suffers, from our point of view, from some limitations: firstly, the clinical setting, which may not be representative of the reality of intensive care in its entirety, and secondly, the state of consciousness of the studied patients. Awake patients may not be the optimal target for quantitative nociception monitoring: many confounding factors can overlap in fully awake and conscious patient, with the potential to distort NOL values.

This is the main reason why we decide to conduct our study in a general intensive care setting, in deeply sedated and sometimes curarized patients only with critical medical issues (no postsurgical patients). We thought this choice could make our population representative of a broad range of intensive care realities, while the state of deep sedation could highlight the real performances of nociception level index, eliminating one of the main confounding factors, namely, wakefulness of the patients, from the analysis.

We evaluated changes in traditionally adopted pain recognition indices such as BPS, HR fluctuation and SBP rise, along with possible alterations in bispectral index as a marker of sedation depth. Each of the examined indicators showed statistically significant variations from baseline during nociceptive stimulation. However, the strength of the association between parameter variation and painful stimulation differed significantly for NOL. NOL exhibited greater variation compared to BPS, HR or SBP.

*NOL-BPS relationship.*

Behavioural pain scale (BPS) is a well-validated tool for the monitoring of pain in patients unable to self-report their algic condition. However, as outlined by PADIS guidelines, BPS is not applicable in the context of deep sedation (RASS ≤  − 4) or curarization.

This is one of the various reasons we decided to split our population in two subgroups: patient under continuous infusion of neuromuscular blocking agents and patients who were not administered NMBA.

Non-curarized patients were subjected to moderate sedation, making BPS a reliable and viable instrument to monitor patient responses to painful stimulation, in accordance with international guidelines [13, 14].

Curarized patients were arbitrarily assigned a BPS value of 3 during data collection, as this is the operator perception of the patient’s state. A relaxed, motionless patient who tolerate with great compliance mechanical ventilation is the misleading impression nursing and medical staff obtain when evaluating heavily sedated curarized patients.

However, it is essential to be aware that nociception processes may still be present despite the absence of observable behavioural responses. Ongoing nociception elaboration in spite of deceitful behavioural response actually is the founding rationale for nociceptive monitoring. The dichotomy between noticeable reaction to pain and the invisible processing of nociceptive information highlights the extreme need for pain monitoring objective tools.

As pointed out in paragraph 4 of the “Results” section, collected values on the pain behavioural scale were not included in the statistical analysis, since the scale is not applicable to pharmacologically paralyzed and deeply sedated patients. Our results, therefore, do not suffer from these arbitrarily given assessments of BPS.

Still, our goal was to make a representation as true as possible of the operator’s perception in front of the patient, and this is the rationale for assigning a BPS value of 3 points in the heavily sedated and curarized subpopulation.

In the non-curarized subgroup, it is interesting to note that BPS exhibits a weaker relationship with nociceptive activation. The limited association between NOL and BPS can be explained by several hypotheses.

Firstly, BPS is the only examined index that may be influenced by inter-operator variability, as the final score is subject to a certain degree of subjectivity. It cannot be ruled out that in some cases, the assigned BPS values were inappropriately too low. Secondly, the characteristics of the studied population may have hindered proper pain assessment using BPS. Deep sedation, with or without neuromuscular blockade, makes it difficult to accurately judge the scale items. This aspect was one of the fundamental assumptions of our study and further supports the idea that objectively assessing pain in deeply sedated patients is challenging.

However, it is worth noting that even in the study by Gélinas and colleagues, no relationship was found between NOL and the behavioural scale, which in that case was the *Critical-Care Pain Observation Tool* (CPOT). The explanation provided by the authors is intriguing and aligns with the premises of our study. Gélinas attributes the lack of correlation between these two assessment tools to the fact that NOL and CPOT measure different components related to pain. CPOT focuses on expressive behaviours associated with pain, while NOL quantifies physiological parameters involved in the nociception process. Additionally, the aforementioned study also demonstrates the absence of a relationship between anxiety and NOL, further highlighting the distinct areas of application for NOL. Once again, NOL does not appear to be influenced by psychological manifestations of pain experience.

NOL did not show a relationship with BPS for either endotracheal aspiration or pronosupination. However, there is a correlation between NOL and BPS specifically in the context of nursing.

One possibility is that nursing interventions led to an awakening from sedation, enabling a more accurate and reliable psychometric evaluation. Nursing, in fact, represents a persistent nociceptive stimulation, distinguishing it from the other considered painful stimuli. Supporting this hypothesis is the statistically significant association between BPS and BIS during nursing, suggesting a reduction in hypnotic depth. Conversely, there is no statistically significant correlation between NOL and BIS, indicating that the two indices investigate different aspects. It is plausible that the simultaneous increase in NOL and BIS, accompanied by higher BPS scores during nurse assessment, was managed solely by increasing sedative drug infusions by the nursing staff. In this case, the decrease in BPS score would not be attributed to effective nociception control but rather to a deepening of sedation and, consequently, a distortion in correctly interpreting the items on the behavioural scale. However, this hypothesis requires further analysis for validation, and the study was not originally designed to explore this thesis.

### NOL-neurovegetative signs relationship

Our analysis revealed an association between changes in neurovegetative parameters and nociceptive stimulation. However, the strength of this link is considerably weaker compared to the observed association with NOL. The Pain, Agitation, Delirium, Immobility, and Sleep (PADIS) guidelines emphasize the inadequacy of neurovegetative signs as indicators of pain in critically ill adults and recommend using them only as cues to prompt further assessment using appropriate and validated methods [14].

Our data confirms the validity of this recommendation, and they further emphasize the nonspecific nature of neurovegetative signs, which should be regarded as warning signs of the presence of pain rather than a reliable means of quantifying ongoing nociceptive excitation. We consistently observed a lack of association between NOL and neurovegetative signs for each painful procedure considered, regardless of the duration or intensity of the stimulation. The variation in neurovegetative parameters during painful stimulation, coupled with their complete absence of correlation with NOL, suggests low sensitivity and specificity of neurovegetative signs as pain indicators.

The subgroup analysis revealed some differences between curarized and non-curarized patients.

In the non-curarized subpopulation, both systolic blood pressure (SBP) and heart rate (HR) showed a statistically significant relationship with painful stimulation. Although the association with NOL was more pronounced, changes in SBP and HR from baseline appeared to recognize nociceptive activation to a certain degree. In contrast, in the deeply sedated and curarized group, neurovegetative signs did not correlate with NOL, and there was no statistically significant change from baseline during painful stimulation. Once again, we emphasize how relying on neurovegetative signs for pain identification can be misleading, particularly in the subgroup of sedated and curarized patients where accurate assessment is most crucial.

It is worth noting that we intentionally included patients in our dataset who had arrhythmias (atrial fibrillation plus one patient with fixed pacemaker-induced heart rate) as well as microcirculation alterations (peripheral vascular disease and diabetes). These pathologies are recognized as contraindications to NOL monitoring in the published literature and pose a major challenge in the widespread use of this technology in the complex reality of critical care wards.

In our experience, NOL performed well in this subset of patients, and we encountered no significant difficulties in signal detection or index interpretation.

We hypothesize that as long as the photoplethysmographic wave is readable from the sensor and a rhythmicity of the heart beat is maintained (e.g. pacemaker-driven pacing or atrial fibrillation with preserved and rhythmic QRS spacing), the NOL algorithm can produce a reliable value, regardless of the underlying medical condition. However, this conjecture requires further validation to be sustained, and it currently represents an intuition from the authors that still needs further development.

### NOL-BIS relationship

A significant part of our efforts was dedicated to evaluating the relationship between sedation depth, as measured by the bispectral index (BIS) and level of nociception, assessed using the nociception level index (NOL).

One of the main assumptions of instrumental pain assessment methods is the distinct separation between the fields of hypnosis and analgesia.

In our sample, both BIS and NOL exhibited statistically significant variation in response to painful stimulation. However, the changes in BIS values associated with pain were only marginally significant, indicating a weak connection with the painful event. Conversely the variations in NOL from baseline showed a much stronger association with painful stimulation. Additionally, the mean bispectral index values in the total examined population suggested a moderate depth of sedation. Consequently, some patients may have experienced a reduction in sedation level during painful stimulation, which was reflected by increases in BIS values. This speculation finds support in previous research on pain-related cortical excitation during anaesthesia induction. During this stage, the depth of hypnosis may still be suboptimal and not sufficiently stable [37–40]. In a recent review on the topic of nociception monitoring, the potential of detecting algogenic stimulation through depth-of-anaesthesia monitoring is highlighted. However, the authors caution against the risk of misinterpreting the numerical values obtained from processed-EEG devices in the presence of an electromyographic signal [4]. Therefore, it is conceivable that EMG interference could have distorted the BIS values, making them appear higher than they actually were during painful stimulation, leading to deceptive results.

The relation between the hypnotic state and analgesic level was investigated, using NOL increases as a reference for proper nociception recognition. We were confident in NOL’s ability to detect nociceptive excitation, supported by previous validation of NOL as a reliable and specific tool for monitoring nociception.

In the entire population, there was no correlation between NOL and BIS, demonstrating the independence between the two aspects of hypnosis and analgesia.

In the subpopulation of non-curarized patients, there was a statistically significant association between NOL and BIS. This association was no longer present in the population subjected to NMBA infusion. BIS values in the NMBA group indicated a deeper sedation level, both at baseline and after stimulation, compared to the non-curarized group. The NMBA infusion also suppressed potential EMG interference that could have affected EEG trace processing. On the other hand, in the non-curarized group, BIS values were set higher, suggesting lighter sedation levels and/or electromyographic interference. It is possible that the painful stimulation caused at least partial cortical activation in the non-curarized subpopulation, leading to an increase in BIS. Additionally, pain-induced eyebrows frowning or facial grimaces may have caused EMG interference, altering the processing of the EEG signal.

Taken together, our results align with the theory and support the clear distinction between analgesia and hypnosis. This underscores the importance of utilizing separate and appropriate monitoring tools for these two domains. Based on our findings, it is evident that depth of anaesthesia monitoring devices cannot serve as substitutes for pain monitoring technologies.

### NOL: Instrumental monitoring of pain for patients unable to self-report

NOL serves as a reliable and effective monitoring tool, particularly in situations where the monitoring options are scarcer. Furthermore, NOL demonstrated superior performance specifically among deeply sedated and curarized patients, for whom the need for pain monitoring is paramount. This aligns with the underlying concept of NOL monitoring. In the state of deep sedation, patients do not consciously experience pain. Anaesthesia hinders cortical processing of pain, rendering it solely an organic phenomenon devoid of subjective psychological implications. What can be measured are the systemic manifestations of the pain-induced stress response. Within the studied sample, the nociception level index has proven its ability to accurately identify and quantify the pathophysiological reactions to painful stimulation, thereby providing valuable information to guide clinical practice.

While NOL may not be the definitive solution to the pain monitoring challenge in critical care settings, it undoubtedly represents a promising device that can shed light on an otherwise obscure yet fundamental aspect of patient care.

In the study by Gélinas et al., the adoption of NOL in awake cooperative patients admitted to the postsurgical ICU after elective cardiac surgery resulted in a call for further validation of this index in a broader critical care context, including deeply sedated and curarized patients [32]. Similarly, in a recent review on pain monitoring in the ICU, Chanques and Gélinas concluded their examination with unanswered questions regarding instrumental nociception assessment [15]. Even the PADIS guidelines acknowledge the potential of these new monitoring devices while emphasizing the need for further research to clarify their true benefits in the critical care environment [14].

Our study provides partial answers to these unresolved questions, fully integrating the NOL index into the category of ICU pain assessment methods.

### Limits

Our study has several limitations. Firstly. the monocentric design of our research may have influenced our results, as it reflects the practices and habits specific to our ICU setting, which may not be applicable to all ICUs.

Secondly, the number of enrolled patients was small, and it is possible that a larger study population would have provided additional information or potentially led to different results.

Furthermore, the retrospective nature of the study does not allow to draw definitive and unequivocally conclusions about NOL potential in the ICU setting. Although the results obtained are encouraging, we are aware that the descriptive character of the study is itself a limitation.

Thirdly, our study did not include certain patient categories, particularly those admitted for primarily neurologic pathology, trauma or cardiac surgery. Therefore, caution should be exercised when extrapolating our results to the entire critical care population.

The promising results of the retrospective study call for a wider prospective study to include an even more heterogeneous population.

In terms of data collection, we relied on punctual values rather than trends for the purpose of statistical analysis. However, it is widely recognized that trends in parameters are more informative than individual values at a specific time. To address this limitation, we considered mean values within a 30-s time interval before and after noxious stimulation.

Similarly, although the interpretation of EEG raw trace is considered superior to relying solely on the isolated BIS number for assessing sedation levels, we were limited to quantitively comparing data using only BIS numerical values and not the entire electroencephalographic registration. It is important to note that the adimensional numeric value produced by the BIS proprietary algorithm has inherent limitations, and continuous raw trace interpretation can compensate for most of these shortcomings [35, 36, 41]. Therefore, qualitatively EEG analysis may yield different results regarding the controversial BIS-NOL relationship.

We did not conduct an analysis based on pharmacological classes or distribute our sample according to the different types of sedative agents used. However, this does not signify a flaw in the study design, as the sedation level was normalized based on BIS values. Standardizing different pharmacological profiles with instrumental assessment values could actually help generalize our findings to critical care settings where sedative agents different from those used in our study are employed.

## Conclusions

NOL stands as a promising device for pain assessment in the critical care setting. Our study seems to demonstrate its viability in a medical ICU population.

In the studied population, NOL accurately detected noxious stimulation and showed its best performance precisely in the clinical context where reliable pain assessment methods are most lacking.

Our research also confirmed that sedation and analgesia belong to two separate spheres, and as a result, they need to rely on distinct monitoring instruments for their correct assessment.

Further studies are needed to validate our findings on a larger scale and to evaluate NOL’s performance in other categories of patients, such as those with polytrauma or primary neurologic pathology.

## Data Availability

No datasets were generated or analysed during the current study.
